# Unraveling mechanisms of human brain evolution

**DOI:** 10.1016/j.cell.2024.08.052

**Published:** 2024-10-17

**Authors:** Madeline A. Lancaster

**Affiliations:** 1 MRC Laboratory of Molecular Biology, Cambridge, UK; 2 Cambridge Stem Cell Institute, University of Cambridge, Cambridge, UK

## Abstract

Evolutionary changes in human brain structure and function have enabled our specialized cognitive abilities. How these changes have come about genetically and functionally has remained an open question. However, new methods are providing a wealth of information about the genetic, epigenetic, and transcriptomic differences that set the human brain apart. Combined with *in vitro* models that allow access to developing brain tissue and the cells of our closest living relatives, the puzzle pieces are now coming together to yield a much more complete picture of what is actually unique about the human brain. The challenge now will be linking these observations and making the jump from correlation to causation. However, elegant genetic manipulations are now possible and, when combined with model systems such as organoids, will uncover a mechanistic understanding of how evolutionary changes at the genetic level have led to key differences in development and function that enable human cognition.

## Introduction

Perhaps the most striking trait of *Homo sapiens* is our cognitive ability. Humans are the only species on earth to have developed an impressive array of technologies that enable us to explore all corners of the planet and even the outer reaches of the solar system. No other species has acquired the recursive language that enables us to exchange complex ideas and plan for the future,^1^ and nowhere else in the animal kingdom would one find societies with the same degree of complexity and cooperativity.^2^ Although these behavioral outcomes rather obviously set our species apart, what is less clear is how human cognition differs at the biological and mechanistic level to enable these abilities.

Until recently, the only way to examine this question was through comparative descriptive studies, such as neuroanatomical comparisons across species. These studies have enabled truly elegant hypotheses of how human brain structure and function may be different and how such differences have come about developmentally and evolutionarily. Some such hypotheses, such as the radial unit hypothesis put forward by Pasko Rakic over 30 years ago,^3^ have stood the test of time. Yet, when it comes to the human brain, hypotheses about how differences unique to our species may have arisen have remained just that, hypotheses. This is because it is incredibly difficult, if not impossible, to perform the types of functional perturbations in the human context that would be needed to go from correlation to causation. Thus, while theories abound, mechanisms are still largely elusive. However, as described in several recent outstanding reviews,^4–8^ an increasing number of cellular and genetic features have been identified that are unique to our species. This review seeks to place this newfound insight within the context of animal nervous system evolution and highlight how new methods will enable a more complete, mechanistic understanding of human brain evolution.

## The Evolutionary Foundation of the Human Brain

For many decades, it was thought that human cognitive abilities such as language, self-awareness, and higher-order thinking were only found in humans. That viewpoint has changed significantly in more recent years, with observations that many animals can in fact demonstrate self-awareness and even theory of mind, the ability to attribute mental states to others. For example, dolphins can recognize themselves in a mirror^9^ and chimpanzees have been demonstrated to use their own experiences to infer another individual’s mental state and anticipate their actions,^10^ demonstrating theory of mind. Several animals can also communicate using symbolic expression,^11^ with some nonhuman apes able to construct rather convoluted phrases of several words.^12^ These examples demonstrate that the foundation of human cognition does not appear to be unique to humans and at least certain aspects can be found in various other animals. An evolutionary perspective may therefore be particularly informative in untangling the origins and biology behind human cognition.

### Origin of the brain

**Figure 1:**
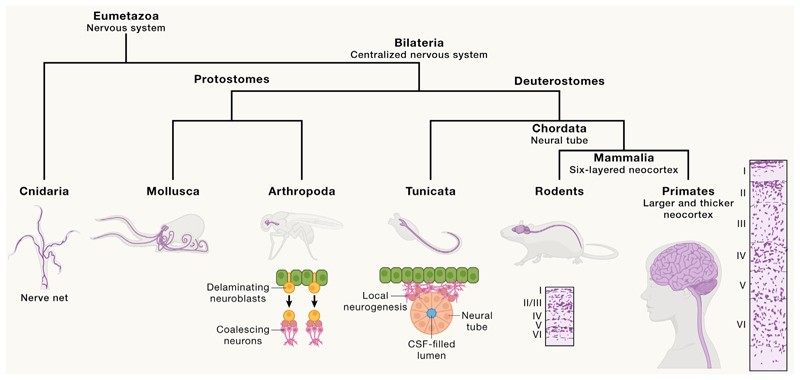
Evolution of the brain Phylogenetic tree (branch lengths not to scale) of selected major metazoan phyla with nervous systems,^14^ illustrating key evolutionary innovations and illustrations of representative organisms: *Hydra* (cnidaria), octopus (mollusk), *Drosophila* (arthropoda), tunicate larva (tunicata), mouse (rodent), and human (primate). Illustrations shown below of differences in neurogenesis in arthropods (left) compared with chordates (right).^15^ Also shown are illustrations of a cross-section of the gray matter of mouse and human cortex, based on the data from Beaulieu-Laroche et al.,^16^ showing the six layers. Mouse and human brain, as well as cross-sections, are shown to scale.

The nervous system first evolved in relatively primitive metazoans and can be found in most animal phyla, including comb jellies, cnidaria, invertebrates, and vertebrates^13^ (Figure 1). Within these clades, nervous tissue varies in its organization and complexity in ways that hint at when and how particular features evolved. Comb jellies, for example, possess a simple nerve net, but unlike in other animals, it is composed of a continuous syncytium instead of independent neurons and synapses.^17^

This is a rather surprising turn of events, as it reveals that Golgi’s reticular theory of the nervous system^18^ is in fact correct for the ctenophores, which likely evolved a nervous system independently. Cnidaria, such as *Hydra* and sea anemone, which have a common ancestor with vertebrates and invertebrates, also have a distributed neural net^19^ but in this case made up of independent neurons and synapses, suggesting that true neurons may have first evolved in our common ancestor with cnidaria. A centralized nervous system, as in a ganglion or brain, can be found across bilateria, including protostomes such as arthropods and mollusks, and deuterostomes, including vertebrates, suggesting that it first arose in our common ancestor with worms and cephalopods. This structural change opened up hierarchical network topologies, enabling information integration and higher-order processing. Thus, impressive cognitive and behavioral abilities can be found in a variety of diverse bilaterians. For example, octopuses have a range of behaviors that many would consider to be evidence of intelligence, including tool use,^20^ long-term memory,^21^ and curiosity.^22^ But not all mollusks exhibit these abilities, and what seems to set octopodes apart is their impressive number of neurons, standing at roughly 500 million,^23^ which is almost 10 times the number in a mouse brain and the most of any invertebrate. Neuron number thus seems to correlate with cognitive ability, even in very distantly related organisms. Given that neurons are the computational units of the brain, it is perhaps not too surprising that differences in neuron number and nervous system size are a common theme in brain evolution.

Turning to chordates, including vertebrates, the major nervous system evolutionary event in this clade was the origination of a neural tube, the anlage that gives rise to the spinal cord and brain.^24^ Unlike other bilateria, where the central nervous system (CNS) develops from coalescing neurons that form ganglia, the CNS of chordates forms from a closed tube of epithelium that spans the anterior-posterior axis and sets up the blueprint of the brain and spinal cord before producing neurons. The advent of the neural tube brought with it two major innovations: the neurogenic neuroepithelium and cerebrospinal fluid (CSF) (Figure 1). Both represent major deviations from the developmental strategy found in other bilaterians. The evolutionary advantage of CSF for those first chordates is unclear but may have related to providing an internal fluid pressure or perhaps roles in chemical sensation.^25^ The evolutionary advantage of the neuroepithelium is more evident, as it provides a founder stem cell pool from which to draw while maintaining the structural blueprint of the brain. This epithelial nature is carried forward in the radial glia, the neural stem cells of the vertebrate brain, which maintain apicobasal polarity and act to guide neuronal progeny to their destinations in an organized fashion. Thus, the evolution of a rather simple structure, the neural tube, brought with it a new degree of organization of the CNS, providing the blueprint that would enable future innovations in brain macrostructure.

### Vertebrate brain evolution

The vertebrate brain is composed of three main regions: the prosencephalon (forebrain), the mesencephalon (midbrain), and the rhombencephalon (hindbrain). These are demarcated shortly after closure of the rostral neural tube by a combination of expansion of the neuroepithelium and morphogenetic movements that produce localized enlargements called vesicles,^26^ separated by constrictions called flexures.^27^ These regions then further subdivide into additional vesicles, with the prosencephalon separating into the telencephalon, which generates the two hemispheres of the cerebrum, and the diencephalon, from which the thalamus and retina derive, while the rhombencephalon separates into the metencephalon, which gives rise to the pons and cerebellum, and the myelencephalon, which gives rise to the most posterior portion of the brain, the medulla.

This same basic blueprint is present across vertebrates, from reptiles and birds to mammals, including humans.^28^ As such, it enables all the basic functions—from sensation and movement to learning and memory—that all vertebrates share. This rough blueprint, composed of distinct territories that specialize for a particular set of cognitive abilities, means that each region and the abilities it affords can exhibit different evolutionary changes. For example, the retina is tasked with sensing light across all vertebrates, but in animals whose environment has necessitated greater visual acuity, such as birds of prey, the cytoarchitecture has evolved to enable much greater packing of photoreceptors (several times that of humans) and, in some birds, the evolution of novel photoreceptors to enable greater color discrimination and even detection of UV light.^29,30^ However, such evolutionary change does not occur in a vacuum, and in order to confer an advantage at the behavioral level, it must involve parallel changes in higher-order processing centers of the brain. Indeed, recent studies have demonstrated that birds have a high neuronal density in the telencephalon,^31^ comparable with primates, and certain avian species of corvids and parrots actually surpass primates in terms of neuronal density. These species have also been shown to exhibit advanced cognitive abilities such as tool use.^32^ This demonstrates again a tendency toward increased neuron numbers in animals with greater cognition. It is tempting to speculate that the epithelial nature of the vertebrate brain, being thus connected through a common tissue of origin and maintaining that connection throughout development, may have enabled independent evolutionary change of brain regions to allow for specialization, while at the same time permitting co-evolution of related brain regions.

### Mammalian brain evolution

The major innovation of mammalian brain evolution is the appearance of the cerebral neocortex, the largest part of the dorsal telencephalon, or pallium, of mammals. All amniotes have a pallium, but the mammalian cortex exhibits a unique cellular organization, or cytoarchitecture, with neurons organized in six layers^33^ (Figure 1). In contrast, birds, for example, have a pallium that is instead organized into nuclei, with clusters of interconnected neurons.^34^ This organization is also found elsewhere in the brain, for example, in the thalamus, where nuclei form local circuitries to perform specific tasks.^35^

These differences in cytoarchitecture are not simply oddities but reflect important differences in functional network topology. For example, the avian vocal nuclei are involved in vocalizations and connect to basal ganglia as part of a loop back to the pallium, which is thought to be key to their extensive vocal learning.^34^ Likewise, the six-layered neocortex reflects a specific topology, with deeper-layer neurons projecting their axons to targets outside the cortex while superficial neurons project their axons to other targets in the cortex, sometimes crossing hemispheres to reach the other side of the brain.^36^ Like the vocal nuclei of birds, there are cortical to basal ganglia connections that form a loop and are similarly involved in motor learning.^34^ However, the layered architecture means that similar topology can be seen in repeating units, called cortical columns or modules, over the cortex, with different cortical areas being broadly dedicated to particular cognitive demands, for example, visual processing in the occipital lobe.^37^ This change in organization may have expanded the local processing capability by introducing additional layers, while at the same time enabling more distributive processing, which is thought to be an important part of higher-order cognition.

### Primate brain evolution

As we move to more recent evolutionary divergences, the differences naturally become less pronounced, but not any less important. The primate brain is a rather typical mammalian brain in many ways. It too exhibits a six-layered cortex, with areas dedicated to particular processing needs, including visual perception, motor control, and learning and memory. But a closer examination of the cytoarchitecture reveals more expanded layers compared with the mouse brain. For example, in the primary visual cortex, the overall thickness of the gray matter composed of neuronal cell bodies is roughly 2.5 times that of the mouse, and this expansion seems to disproportionately affect the more superficial layers, which are expanded more than 4-fold^38^ (Figure 1). Add to this the fact that primates exhibit an increased neuronal density^39^ and it becomes evident that one of the major primate innovations was an expansion in cortical neurons. This expansion is likely an important contributor to the advanced cognitive skills of primates, including their sophisticated social skills. Thus, again, evolutionary expansion in neuron number seems to be an important theme in animals with more advanced cognition.

## Hints at Human-Specific Neurobiology from Comparative Studies

Although various studies in animals have revealed shared evolutionary and developmental mechanisms, identifying processes unique to humans is rather more difficult. However, again, evolutionary comparisons are informative, and in this case comparisons with our closest living relatives can reveal the specializations that set humans apart from other primates. Traditionally, such comparisons have necessarily relied on descriptive approaches, including neuroanatomical and behavioral studies. As with other comparisons, it is difficult to find a behavioral or anatomical feature that is truly unique to humans in a qualitative sense. For example, baboons in the wild are naturally effective communicators, using facial expressions and vocalizations to share information about threats and food sources.^40,41^ Likewise, chimpanzees and bonobos, our closest living cousins, communicate naturally in the wild^42^ and can be trained to use an impressive number of symbolic gestures and to understand human language.^12^ Apes have also been observed using tools in the wild and even training subsequent generations,^43^ much as humans do.

### Comparative neuroanatomy

Like behavior, brain anatomy exhibits little in the way of qualitative differences in humans compared with other primates. Even when it comes to abilities often attributed only to humans, like language, the neuroanatomical features necessary for these abilities, in this case Broca’s and Wernicke’s areas, are found in other primates^44^ and likewise seem to be important for communication.^45^ Similarly, the prefrontal cortex (PFC), an important site of higher-order thought processes, is found across primates and rodents; however, the primate PFC seems to be more elaborate, with a region called the dorsolateral PFC, as well as a granular layer 4 within the PFC.^46^ Along these lines, spatial transcriptomic analysis of the macaque cortex revealed primate-specific cell types within layer 4 not found in rodent,^47^ and a comparison of human and mouse cortex using another spatial technique called multiplexed error-robust fluorescence *in situ* hybridization (MERFISH) revealed human differences in cell-cell interactions.^48^ Furthermore, recent single-cell sequencing revealed differences in transcription factor expression, most notably the broader distribution of *FOXP2* expression across layers 3–6 in primates, whereas this gene is restricted to layer 6 in other mammals.^49^ Notably, *FOXP2* was originally identified in a large family with a speech and language disorder.^50^

These findings highlight the existence of numerous differences between primates and rodents, which are often captured in comparisons between humans and mice. However, comparisons between humans and other primates often find more similarities than differences. For example, primate differences in *FOXP2* neural expression were shared with human,^49^ and while there were certain differences in expression patterns, particularly in microglia, an immune cell type of the brain, there were no human-specific neuron types not present in at least one other primate.^49^ This is consistent with previous studies, which have shown that while several instances of potential human-specific neuron types have been proposed, for example, von Economo neurons,^51^ further investigation has revealed these cells to be present in other mammals.^52^ Recently, a special inhibitory neuron type called the rosehip neuron was identified in the human brain,^53^ but it remains to be determined whether it is human-specific or rather shared with other primates, as only a comparison with mouse has so far been carried out. Thus, it is not yet clear how much the cognitive basis for human intelligence may come from uniquely acquired regions, cell types, and other qualitative differences, or from quantitative differences in shared cell types.

Overall, something about the degree and complexity of thought may be what sets humans apart. For example, for all their training, nonhuman apes cannot construct recursive, semantic sentences in which information is embedded within another representational phrase.^54^ This added complexity, combined with the sheer number of symbols (words) humans can learn, makes for infinite possibilities. Furthermore, while symbolic communication is found throughout the animal kingdom, no other animal, including other apes, has shown the same endless curiosity and propensity to ask questions that comes naturally to a human child.^55^ Thus, what seems to set human cognition apart is the degree of thought, curiosity, and communication, and the combination of all these skills at once. Yet how such differences may have arisen evolutionarily, and the biological mechanism for this increased complexity, remains to be determined.

**Figure 2:**
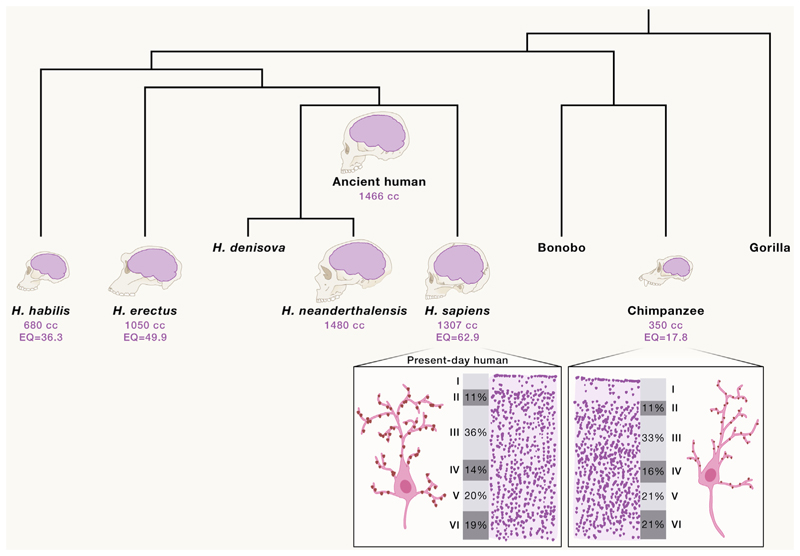
Evolution of human-specific brain features Phylogenetic tree (branches not to scale) of the hominoids, including ancient hominin relatives and the great apes. Shown below selected species are illustrations of brain and skull shape, cranial capacity (cc, cubic centimeters), and encephalization quotient (EQ). *H. habilis, H. erectus*, and *H. neanderthalensis* morphology data from Bruner and Beaudet,^60^ present-day and ancient human morphology data from Neubauer et al.,^61^ and chimpanzee morphology data from Gómez-Robles et al.^62^ EQ and volume data from Williams,^58^ DeSilva et al.,^63^ and VanSickle et al.^64^ Below the human and chimpanzee are shown illustrations of the cortical thickness and layering of cortical area VP, based on data from de Sousa et al.,^65^ with the average of the relative proportions of layers in cortical areas V2 and VP.

There are hints from comparative studies with our closest cousins, the other great apes. Although humans have a rather typical primate brain in terms of its organization and neuronal density,^56^ it is anything but typical in terms of absolute neuron number. Thus, even though the number of neurons is typical for its size,^57^ that size is precisely what sets it apart. On top of that, humans have a rather diminutive body size, much smaller than would be predicted for our brain size. Across mammals, brain size and body size correlate nicely,^58^ suggesting that there are certain hard-wired developmental rules to ensure the two are matched. Yet, humans seem to break that rule. The human encephalization quotient, essentially a measure of how well the brain and body size match the general trend across mammals, is the largest of any mammal^59^ (Figure 2). Thus, while the number of neurons in the human brain is predictable given its size, human brain size is atypical. How body growth and brain growth have become so uncoupled in human development is a key question,^66^ the answer to which could reveal mechanistic insight into how this feature has come about.

Looking closer at the cytoarchitectural level, building on the primate-acquired expansion of cortical layers, great apes exhibit even thicker cortical gray matter compared with monkeys like macaques.^67^ This suggests an evolutionary event in apes that has enabled even more extensive cortical connectivity through elaboration, especially of superficial intracortical connections. Expansion in cortical neuron number seems to have continued in the human lineage, leading to roughly 2-fold more neurons than other great apes.^68^ This equates to an increase of roughly 8 billion neurons. That is a massive increase. To put this into perspective, if we divide that number by the time it takes to complete neurogenesis (approximately 112 days^69^), the result is almost 3 million per hour. Given that cell cycle dynamics within apes are not thought to be dramatically different, such a staggering increase likely relates to a combination of increased numbers of progenitors and extended neurogenic period.^69^ Despite this overall increase, cortical layering is unchanged in humans^38,65^ (Figure 2), and recent comparative transcriptomics reveals no difference in relative proportions and distribution of cell types in cortical layers of great apes.^70^ This suggests that, instead of increasing the number and diversity of neurons within cortical columns or modules, rather the number of modules has increased, with increased number and diversity of cortical areas.^37^ Because such modules are often dedicated to particular processing tasks, such an increase may allow for more elaborate processing capability and be an important factor in our increased cognitive capabilities.

Finer resolution comparisons at the single-cell level have also uncovered differences in the degree of connectivity. For example, compared with rodents, human cortical neurons exhibit much more elaborate dendritic branching and larger spines,^71^ the postsynaptic swellings that undergo remodeling during learning and memory. In addition, due to the large expansion of supragranular neurons in primates, there are increased cortico-cortical connections within the human brain compared with the rodent.^72^ Even compared with apes, human neurons are more branched and dendrites are longer than chimpanzee in all cortical areas examined^73^ (Figure 2). This suggests that, on top of the increased neuron number, the human cortex is also more interconnected through its much more elaborate cortical neuronal properties.^72^ The combination of 4-fold more dendritic spines per neuron^73^ with the increase in neuron number equates to a total increase in cortical synapse number of roughly 14 trillion, which is more than the number of galaxies in the universe. This is particularly impressive, considering how closely related humans and chimpanzees are. It is hard to ignore such a massive increase when considering potential factors contributing to our advanced cognition, and recent studies of human intelligence have revealed a correlation with superficial cortical thickness and neuronal morphology.^72,74^

### Comparative omics

The last 20 years has seen an explosion of new technologies that have revolutionized molecular and cell biology and opened the door to evolutionary studies that were previously impossible. Next-generation sequencing has made it possible to easily and cheaply sequence the genome of any living species.^75^ This has spurred new efforts to catalog the genomes of a diverse set of animals, with the Darwin tree of life project even aiming to sequence all eukaryotes in Britain and Ireland.^76^ These impressive efforts will provide a fountain of data for comparative genetics. By comparing the genomes of related species with divergent characteristics, one can develop hypotheses about which genetic changes may be responsible for those characteristics and how they may have evolved.

When it comes to brain evolution, comparative genetics has unveiled a range of changes in the human genome that are promising leads. The difficulty arises in wading through the many genetic changes to uncover which are most likely responsible for our divergence in phenotype. First, although the human genome is highly similar to that of other apes, exhibiting only ~4% divergence^77^ (contrasted with the mouse-rat difference^78^ of more than 20%), this still equates to more than 35 million single-nucleotide variations and over 90 Mb of genomic rearrangements, including insertions and deletions. Even on a chromosomal level, humans have acquired a major rearrangement with the fusion of two chromosomes to give rise to chromosome 2, which in other apes is split over chromosomes 2a and 2b.^79^

The second difficulty comes in discerning which genetic changes may have contributed specifically to neurobiological differences. Phenotypic differences in humans are not only present in the brain. Indeed, other tissues and cell types are likely under much stronger selective pressure, such as reproductive organs and cells, and immune cells involved in a never-ending arms race with viruses and other pathogens. Thus, a given genetic change in a mitotic regulator could be responsible for increased neuron numbers but equally likely could be important for increased sperm production. For example, in humans, the gene *HYDIN* has been specifically duplicated so that humans have an additional *HYDIN2* gene not present in other apes.^80^ The parental gene is important for cilia/flagella function and for sperm motility,^81^ but *HYDIN2* seems to be more broadly expressed in the brain, suggesting that it may have been co-opted for a new role in a different context. However, further mechanistic insight would be needed to determine what effect this genetic change has on the phenotypic level.

A solution that addresses both these difficulties is the integration of comparative genetics with transcriptomics and epigenetics. By surveying the expression signatures of various organs, one can begin to predict where various genes are likely to play important roles and thus where their genetic changes may have an influence. This is particularly true for the vast numbers of genes of unknown function, or the unknome.^82^ Furthermore, by surveying across species and performing comparative multi-omics, we can begin to interpret the vast numbers of noncoding changes and develop hypotheses about the roles such changes may have on protein expression or isoform variation.

Add to this the recent development of single-cell technologies and we are at a point in history where, for the first time, we can characterize in depth the genetic, epigenetic, and transcriptomic signatures of millions of cells across many organs and species. It is a truly invigorating moment in scientific history. Recently, a series of studies produced a wealth of data on the regulatory and expression landscape across different cell types and regions of the human brain as well as nonhuman primate brains.^70,83^ Perhaps the only downside of these exciting new approaches is the sheer quantity of data and finding ways to make sense of the flood of information. But this is a good problem to have, and through careful, statistically controlled comparisons, such studies are beginning to highlight genetic changes with strong evidence for a role in brain evolution.

One limitation of comparative transcriptomics is that, unlike the genome, the transcriptome is not static. Thus, these comparisons require independent samples for the organ or cell of interest and for the given set of species, not to mention the age or developmental stage, which can be much more difficult to acquire than a simple cheek swab or other non-invasive genetic sample. This makes it challenging to perform the extent of comparisons necessary to reliably detect species-specific differences. For example, only comparing two species, such as human and chimpanzee, makes it impossible to know in which species a change in expression was acquired. At the genetic level, it is easy and common practice to examine a range of species and include outgroups, but studies at the transcriptional level are usually less well powered because of these limitations. However, as single-cell technologies further improve and combinatorial barcoding methods^84^ enable more cost-effective ways to scale-up sample sizes, it is likely that broader comparisons across species, time points, and tissues will become more commonplace.

## Methods to Reveal Mechanistic Insight

The combination of comparative neuroanatomy and omics is now providing compelling possible explanations for the evolutionary origin of human brain differences. For example, *FOXP2*, the transcription factor found to exhibit differential expression in primate excitatory neurons and microglia,^49^ and associated with speech and language development,^50^ also exhibits signatures of potential selection at the genetic level. There are several putative human accelerated regions (HARs)—highly conserved regions that in humans exhibit significant change—as well as substitutions that have become fixed in humans but where Neanderthals carry the ancestral allele.^85,86^ Epigenetic comparisons have also uncovered regions of differential accessibility between humans and chimpanzees.^87^ Together, these data suggest the *FOXP2* locus may have undergone substantial selection in humans to enable a divergent expression pattern and potentially influence language acquisition. However, while these comparisons reveal a fascinating set of connected observations, they remain correlative, and without functional studies and genetic perturbations, it is impossible to take candidate genetic changes beyond hypothesis.

For obvious reasons, genetic perturbation in an intact human brain is not an option. Thus, scientists must turn to model systems. Although at first glance it may seem impossible to study human brain evolution in the context of an animal model, elegant studies have recently been carried out using the mouse as a kind of host genetic background in which to test human-acquired genetic changes. For example, the gene *GADD45G* contains a human-specific deletion in an otherwise conserved region (hCONDEL).^88^ *GADD45G* is a putative tumor suppressor gene and is expressed during cortical development, suggesting that such a deletion may influence its activity in this context. To gain insight into the function of this region, and the effect of its loss in the human context, McLean et al.^88^ constructed reporter constructs containing the chimpanzee sequence with the intact region and injected mouse embryos to investigate which tissues showed the strongest activity. Consistent with a potential role in brain development, the hCONDEL near *GADD45G* showed strong activity in the forebrain. Such comparative transgenic assays can thus be highly informative in predicting functional effects of human-acquired genetic changes.

Similarly, cell lines have been used as a testing ground to explore the potential effects of human-specific genetic changes. To date, thousands of HARs have been identified,^89–91^ but whether these represent functional regions is unclear from sequence alone. Likewise, comparative epigenetic studies have identified thousands of regions in the human genome that exhibit increased enhancer activity compared with nonhuman primate or mouse,^92^ and human-specific genetic divergence can also be observed in such regions. In order to assess on a large scale whether these numerous human-acquired genetic changes play a role in activity of the regions in which they lie, massively parallel reporter assays (MPRAs) have been performed in human neural stem cells,^93^ in human and mouse neuroblastoma cell lines,^94^ and in human and chimpanzee neural progenitors.^95^ When comparing the activity of the human sequence with that of the chimpanzee, 30%–60% of tested regions exhibited differential activity, suggesting that the human-acquired genetic changes could be impacting their regulatory roles. However, linking such changes and their activity to their target genes and to their *in situ* functions is much more challenging, and it is likely that only a very small percentage of such changes play important roles in the context of the human brain.

Recently, more advanced *in vitro* models called brain organoids have been applied to these evolutionary questions.^96–98^ Organoids are self-organizing tissues that develop according to intrinsic developmental and morphogenetic programs,^99,100^ similar to their *in vivo* counterparts. As such, they can provide a bridge between simpler two-dimensional (2D) cultures and the *in vivo* brain.^101^ This makes them rather more complex than other *in vitro* models, which can make them more technically challenging,^102,103^ meaning careful quality control and benchmarking are key.^104^ However, these limitations are offset by the fact that they can be generated from human or ape cells and offer a window into otherwise hidden developing tissues. Initial studies with brain organoids focused on disease modeling, with the first such disorder being a genetic form of microcephaly, or brain undergrowth, caused by a mutation in *CDK5RAP2*.^99^ Because organoids recapitulate the tissue context of the developing cortex, an abnormality in spindle orientation could be observed and could explain the undergrowth also seen in the patient-derived organoids. This demonstrates the power of organoids for performing the types of studies normally performed in animal models, but in a human context. Furthermore, the fact that organoids could capture the microcephalic phenotype with a level of brain reduction in line with the difference in brain size between human and chimpanzee points to their potential utility for evolutionary studies.

## Potential Human-Specific Developmental Mechanisms

Many of these newly developed, cutting-edge tools are already beginning to yield exciting new insights—from the earliest stages of brain development through to neuronal maturation and function. These insights from development are now uncovering key differences in the way the human brain grows and matures, revealing why the final product looks so different. By taking a developmental approach, the evolutionary differences are also starting to become clearer.

### Brain expansion

**Figure 3:**
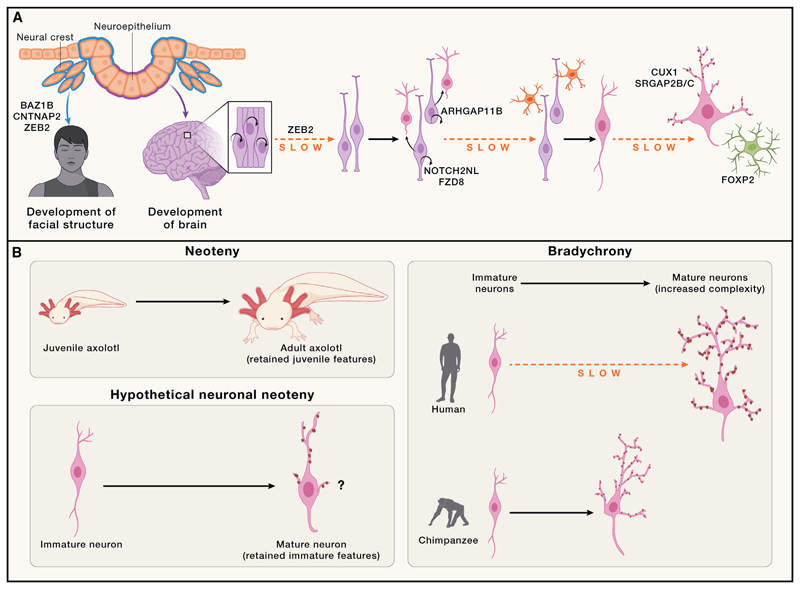
Potential human-specific mechanisms (A) Illustration of human-specific developmental differences. Early neuroepithelium gives rise to neural crest, which contributes to facial structures, while retained neuroepithelium gives rise to the brain. The telencephalon is particularly expanded, with increased proliferative capacity of the neuroepithelium, shown in a magnified view, due to delayed transition to radial glia. Radial glia and basal progenitors, including outer or basal radial glia, are highly proliferative in humans. The transition to astrogliogenesis, represented by orange astrocytes, is delayed, as is the maturation of neurons. A unique subtype of microglia expressing *FOXP2*^49^ is represented by the green cell at the right. Key identified genes and their developmental contexts are also shown. (B) Illustration of the difference between neoteny and bradychrony. Neoteny was originally coined in reference to the axolotl,^106^ which retains juvenile features in adulthood. Below is shown the result of hypothetical neuronal neoteny, which would similarly result in juvenile neurons. Instead, bradychrony results in mature neurons in adulthood, but because the process is slower, the result is increased complexity.

Like the comparative neuroanatomical studies of the adult brain, comparative studies of the developing brain can provide insights into potential developmental mechanisms. Early comparisons with accessible nonhuman primates, like macaque monkey, demonstrated that even at the early neuroepithelial stage, the human forebrain is already larger than that of the monkey, suggesting potential expansion of the founder neuroepithelium as a mechanism for brain expansion.^105^ However, whether such a mechanism represents a human-specific difference or one shared with other apes was unknown, and, therefore, the mechanism for human-specific expansion remained to be determined. Although a comparison with a nonhuman ape, such as chimpanzee or gorilla, during embryonic or fetal stages is not possible due to ethical reasons, organoids can provide an accessible alternative. Comparison of human organoids to chimpanzee and gorilla revealed a more expanded neuroepithelium,^98^ suggesting that not only are neuroepithelial founder cells increased in apes but they are also further expanded in humans. The reason for this expansion seems to be due to a slower transition in human from the faster-cycling neuroepithelium to the slower-cycling radial glial stem cell (Figure 3). Hence, in humans, a larger founder pool is established even before the onset of neurogenesis. Such a change would increase all subsequent progeny and is consistent with the observed increase in cortical neurons.

It would also explain how such an increase could involve no change in cortical thickness, as is seen between humans and other apes, but rather an increase in cortical columns or modules.

To bring these observations to mechanism, genetic insight is needed. Comparative transcriptomics of human and gorilla organoids revealed a number of candidate genes with differential dynamics, consistent with the slowed tempo of neuroepithelial transition.^98^ One in particular, *ZEB2*, seems to be an important regulator of this process, and perturbation of ZEB2 was able to phenocopy the species-specific differences in neuroepithelial transition, suggesting that it may be an important genetic determinant. Supporting this, the *ZEB2* locus contains four HARs and one hCONDEL, a large number of such elements for a single gene. However, further functional studies of the effects of these genetic changes are needed to determine whether they, and *ZEB2*, are really involved in human-specific neuroepithelial expansion.

### Neural crest differences

Concomitant with neuroepithelial expansion, another important morphogenetic process occurs very early in neurodevelopment: the production of the neural crest. The neural crest is a population of multipotent stem cells that emerge from the dorsal neural tube at various sites along the anterior-posterior axis, including the emerging brain.^107^ Neural crest cells arise from an epithelial to mesenchymal transition of specific neuroepithelial cells that delaminate and begin migrating to distant locations (Figure 3). In the case of the cranial neural crest, these cells build the supportive structures of the head, including the skull, cartilage, and certain ganglia, and therefore are the basis of human head and face structure.

As with exploration of brain differences, *in vitro* models can uncover potential mechanisms that distinguish the human neural crest. Comparative epigenetic analysis of cranial neural crest cells differentiated from human or chimpanzee pluripotent stem cells revealed a large number of differentially active enhancers, and transgenic reporters introduced into mouse embryos demonstrated striking differences in where these enhancers were active.^108^ For example, the chimpanzee enhancer near the gene *CNTNAP2* was expressed mainly in the olfactory placode, but the human enhancer showed additional expression in the nasal pit and eye pit, as well as in the telencephalon and the future cerebellum. Many of these enhancers affect genes associated with syndromes involving craniofacial abnormalities, suggesting that these may be key to the evolution of the human face. Similarly, the gene *BAZ1B* is associated with a rare human condition called Williams-Beuren syndrome, which includes craniofacial dysmorphism.^109^ *BAZ1B* is a chromatin modifier that regulates expression of a range of genes important for neural crest development, many of which exhibit signatures of selection in modern humans.

Interestingly, many of the genes implicated in human neural crest evolution also play important roles in brain function and/or have been implicated in neurodevelopmental or neuropsychiatric disorders. For example, Williams-Beuren syndrome is also associated with neurocognitive deficits^109^ and, likewise, *CNTNAP2* is associated with autism and schizophrenia.^110^

Further, many neurodevelopmental disorders exhibit craniofacial abnormalities and several key brain development genes function in neural crest development as well. Along these lines, *ZEB2* is necessary for proper development of the anterior neural crest, and mutations in *ZEB2* cause Mowat-Wilson syndrome, characterized by defects in the enteric nervous system (which is derived from the neural crest), craniofacial abnormalities, and intellectual disability.^111^ Given that the neural crest arises from the same original neuroepithelial primordium as the brain (Figure 3), this overlap may not be a coincidence. One intriguing possibility is that a common molecular mechanism may have driven the evolutionary expansion in brain size and, at the same time, resulted in more diminished craniofacial structures such as the jaw and brow,^112^ compared with other apes and ancient hominins.

### Differences in neurogenesis

Cortical neurogenesis begins when neuroepithelial cells transition to neurogenic radial glia, the neural stem cells of the brain.^113^ There are various products of radial glial divisions, but the most common outcome is a self-renewing asymmetric division giving rise to another radial glial stem cell and a more differentiated daughter cell, usually an intermediate or basal progenitor. Basal progenitors can further proliferate and undergo neurogenesis, thus expanding the number of neurons produced. As development proceeds, neurogenesis becomes more dependent on these basal progenitors, including a population of radial glial cells that become displaced from their apical location and become known as basal, or outer, radial glia.^114,115^ Neurons are produced in a specific temporal order, with deep-layer neurons produced first, followed by superficial-layer neurons. Thus, an expansion of basal progenitors disproportionately affects the production of more superficial-layer neurons.^116^ Indeed, in primates and several other large-brained mammals, there is a dramatic expansion in these basally located progenitors leading to the appearance of a progenitor zone called the outer subventricular zone (OSVZ).^117^ The OSVZ becomes the major site of neurogenesis during mid-neurogenesis in such species, by which point deep-layer neuron production is complete and superficial-layer production has taken over. This expansion in basal progenitors thus explains the thickened cortical gray matter and increased proportion of superficial layers in primates compared with small-brained rodents like mice. But what is the evolutionary genetic mechanism for this expansion, and are there human-specific differences during neurogenesis that diverge from other primates and apes?

Recent studies in various model systems are beginning to shed light on these questions. As with earlier stages, neurogenic stages of nonhuman ape brain development are not accessible, but *in vitro* models can provide a window into this otherwise black box. Early comparisons of 2D neural rosettes differentiated from human, chimpanzee, and macaque cells revealed a faster progression through neurogenesis for macaque compared with ape cells, which was also seen in three-dimensional (3D) organoids.^118^ More recently, single-cell RNA sequencing of human fetal developing brain, macaque developing brain, human organoids, and chimpanzee organoids enabled the cross comparison of different species and the validation of organoids for modeling these processes,^97^ which is especially important for species such as chimpanzee for which developing brain tissue is unavailable. This revealed highly similar cell types and progression across species and between *in vivo* and *in vitro* samples. Although *in vitro* organoids exhibited a small subset of gene expression modules associated with glycolysis, a signature not seen for *in vivo* samples, over 70% of gene expression modules showed high correlation between fetal brain and organoids.^97^ Cross-species comparison revealed a number of differentially expressed genes in human samples, with several factors involved in mTOR signaling, which was increased in human radial glial stem cells, particularly those in the OSVZ. This suggests a potential human-evolved difference in mammalian target of rapamycin (mTOR) signaling in human radial glia, though the genetic basis of such a difference is still unclear.

To uncover such a genetic basis, comparative genomics are providing a window into human-acquired genetic changes, with strong evidence for involvement in neurogenesis. A HAR in an enhancer near the *FZD8* gene was shown to exhibit stronger activity for the human sequence in a transgenic mouse reporter than the chimpanzee sequence.^119^ Taking this a step further to a set of elegant functional studies, transgenic mice overexpressing *FZD8* under the control of the human HAR enhancer exhibited increased neural progenitor proliferation and a subtle increase in neocortical size, showing not only a species-specific difference in enhancer activity but also that this activity has an effect on development of the neocortex *in vivo*. Another HAR with differential activity was identified near the *PPP1R17* gene, which showed primate-specific expression in neocortical basal progenitors.^94^ Overexpression of *PPP1R17* in mouse neural cells led to a prolonged cell cycle, in line with the observed slower cell cycle of primate neural progenitors compared with mouse. However, further mechanistic studies are needed to determine whether and how the human-specific genetic changes at the *PPP1R17* locus influence neural progenitor proliferation.

In addition to evolutionary genetic changes in regulatory regions, several genes have arisen *de novo* in the human lineage through genetic duplication events.^120^ Although the majority of duplicated regions do not produce functional proteins, there are an increasing number of genes that have in recent years been shown to not only produce functional products but also have strong evidence for contributing to evolutionary changes that affect the brain. *TMEM14B* arose in primates specifically, and ectopic expression in the developing mouse cortex leads to increased production of basal radial glia and neurons.^121^ The evolution of apes brought with it additional novel genes, including *TBC1D3* and *CROCCP2*, both of which similarly lead to increased basal radial glial progenitors when ectopically expressed in the mouse developing cortex.^122,123^

**Figure 4:**
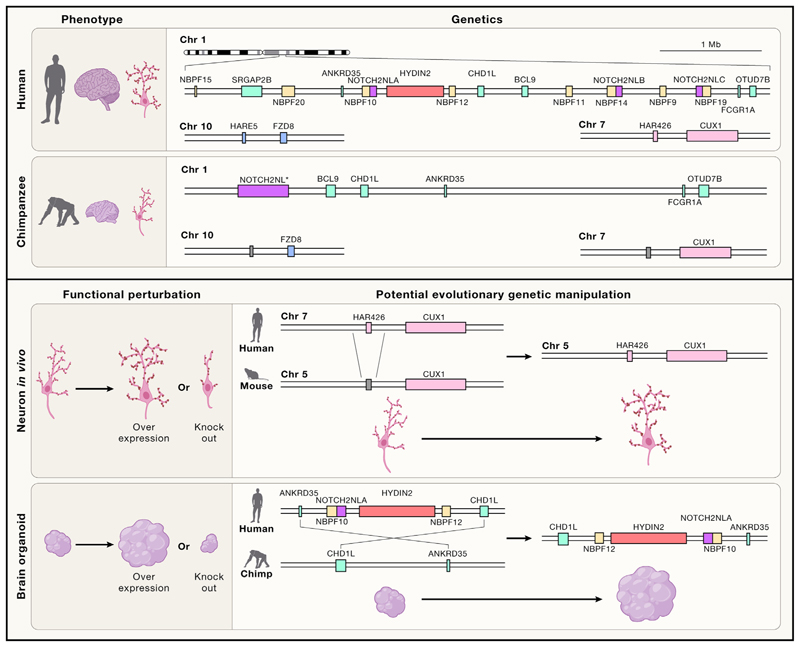
Unraveling mechanism Comparative studies reveal species-specific phenotypic differences, such as brain size and neuronal morphology, which can be correlated with genetic differences. Shown are a few representative human-specific genetic differences correlated with neurodevelopmental differences. A region of chromosome 1q21 is shown, revealing the large number of novel human genes. HARs at the FZD8 and CUX1 loci are also shown as examples. Below are the homologous chimpanzee loci with the ancestral NOTCH2NL duplication resulting in a nonfunctional product (denoted by *). Functional studies, such as overexpression or knockout in model systems provide strong support linking genetics to phenotype. In the future, refined genetic manipulation of model systems, such as organoids or mice, to mimic the evolutionary changes will reveal whether and how human-specific changes have enabled differences in brain development to give rise to the large and complex human brain.

Turning to human-specific genetic duplication events, a region on the long arm of chromosome 1, 1q21, exhibits a large number of duplications (Figure 4). This region is also associated with a set of human conditions involving copy-number variations leading to microcephaly in 72% of those with a deletion, and macrocephaly in 42% of those with a duplication,^124^ suggesting that it may house genes important for brain development. Among the genes duplicated at this site, the *NBPF* genes are the class with the most expansion in humans, comprising approximately 165 human-specific versions.^125,126^ Although the exact function of the proteins encoded by *NBPF* genes is still unclear, several appear to be specifically expressed in human neural progenitors and neurons,^125,127^ and overexpression seems to increase neural stem cell proliferation.^127^

Also, within the 1q21 region, a group of NOTCH-related genes called *NOTCH2NL* have been duplicated to give rise to three novel functional genes in humans, whereas other apes carry a single nonfunctional version^128^ (Figure 4). NOTCH2NL proteins seem to act on delta receptors in *cis* to promote NOTCH signaling and potentiate its proliferative effects.^129^ Indeed, over-expression of NOTCH2NL in the mouse developing cortex, or in human cortical progenitors, leads to progenitor amplification,^129^ while knockout of two of the NOTCH2NL genes in human cortical organoids leads to premature neuronal differentiation.^128^ These findings make a highly compelling case for the involvement of NOTCH2NL duplications in human brain evolution.

Because basal progenitors are so expanded in primates compared with rodents, recent studies have also explored potential roles for human-specific genes in their biology. By comparing the transcriptomic signature of human basal radial glia to those of mouse, *ARHGAP11B* was shown to be specifically expressed in human progenitors.^130^ Overexpression of *ARHGAP11B* in the developing mouse cortex increases the production of basal progenitors and even leads to the local appearance of folding, reminiscent of gyri, suggesting that ARHGAP11 proteins may be involved in primate brain expansion and gyrification. Perhaps even more intriguing, human *ARHGAP11B* ectopically expressed through lentiviral integration in the marmoset, a primate with a small and smooth brain, led to a subtle but significant increase in brain size and the production of a new gyrus.^131^ As in the mouse, basal progenitors were increased and cortical thickness was also increased. Consistent with the role of basal progenitors primarily in later stages of neurogenesis, only superficial-layer neurons were increased. Overall, ARHGAP11B seems to increase superficial-layer neurogenesis through its promotion of basal progenitor proliferation. How this fits with human-specific expansion is still unclear, given that superficial layers are not increased compared with other apes. Thus, further studies are needed to investigate how *ARH-GAP11B* contributes to human-specific phenotypes.

### Glia and other supportive cell types

At the end of neurogenesis, radial glial progenitors switch to gliogenesis, producing astrocytes and myelinating oligodendrocytes. Historically, these cell types have received less attention than neurons, but it is now clear that glia perform not only vital supportive roles but also actively participate in nerve transmission and synapse remodeling to shape the neural circuitry of the brain.

Once thought to be just one population, astrocytes are now recognized as encompassing a variety of molecular and morphological subtypes, and comparative studies in primate and rodent brains are revealing important evolutionary differences in these populations. Human astrocytes are much larger than their rodent counterparts, and additionally include subclasses not present in the rodent brain.^132^ Much of this increase in diversity and complexity seems to have been a primate-acquired trait, shared between humans and nonhuman primates^133^ and playing key roles in primate brain expansion and folding.^134^ For example, interlaminar astrocytes, a population with highly extended processes that contact various neurons across cortical layers, are greatly expanded in number and are larger in the monkey cortex compared with mouse,^135^ a feature shared with humans and chimpanzees. Furthermore, in apes, astrocytes have further diversified with the appearance of varicose projection astrocytes, a morphological subclass only found in hominoids.^136^

Human-specific differences are more subtle, but transcriptome profiling is beginning to reveal important differences in astrocytes compared with our ape cousins. Cerebral organoids and cortical spheroids both revealed a faster switch to gliogenesis in chimpanzee compared with human^96,137^ (Figure 3). To reveal *cis* versus *trans* gene regulatory mechanisms, human and chimpanzee cells were fused to generate allotetraploids, uncovering a human allele bias in a set of astrocyte-associated genes. Furthermore, while the same diversity in astrocyte subtypes can be found in chimpanzee brain, the size and number of certain subtypes seem to be increased in humans.^132^ In addition, *in vitro*-derived astrocytes from human, chimpanzee, and macaque cells revealed a difference in astrocyte size and expression of cell-size-related genes^138^ that may help explain the increase in morphological complexity of human astrocytes.

Oligodendrocytes, the myelinating cells of the brain, are a more recently evolved cell type, present only in vertebrates. Like astrocytes and neurons, they are generated from radial glia, but through oligodendrocyte precursor cells—a class of progenitors that are also maintained in the adult brain and are increasingly recognized to exhibit diverse subclasses.^139^ Recent transcriptomic studies have revealed that these progenitors arise from basal radial glia and that they expand exponentially in the developing human brain to massively increase their numbers.^140^ Furthermore, comparison of human, chimpanzee, and macaque brains revealed a human-specific change in the balance of oligodendrocytes to oligodendrocyte precursors^141^ and a human-specific gene network that sets human oligodendrocytes apart.^142^ Finally, single-cell RNA sequencing of human, chimpanzee, bonobo, and macaque brains^143^ revealed the greatest human-specific differences in molecular signature in astrocytes and oligodendrocyte precursor cells. These findings point to both major glial populations as having undergone key evolutionary changes in the human brain that warrant further mechanistic investigation.

Brain function also depends heavily on cell types and influences that originate from outside the brain. Microglia are an immune cell type highly similar to the tissue-resident macrophages found elsewhere in the body and, likewise, originating from the yolk sac of the developing embryo.^144^ Once microglia take up residence in the brain, they contribute as phagocytic cells, clearing debris and pathogens but also contributing to synaptic pruning. As such, microglia are important regulators of brain circuitry and function. Strikingly, in comparative transcriptomics of the dorsolateral PFC, only one cell type was found to be unique to humans: a subtype of microglia expressing *FOXP2*.^49^ This suggests much broader roles for *FOXP2* than previously thought and, combined with the several human-specific genetic changes, points to the need for further mechanistic investigation in the context of microglia.

Brain function is also heavily dependent on CSF, which itself changes during development as the CSF-producing choroid plexus matures. Although almost nothing is known about evolutionary differences in the choroid plexus and CSF, the recent development of human choroid plexus organoids revealed a number of proteins that seem to be produced and secreted in human CSF but were not detected in rodent or cow CSF.^145^ Further comparative studies are needed in other primates to determine whether there are any human-specific differences in this vital fluid and whether there might be genetic divergence in choroid-plexus-expressed genes.

### Neuronal maturation and function

Turning to neurons themselves, several studies are beginning to highlight potential mechanisms underlying the increased complexity in neuronal morphology and the slower neuronal maturation. Targeted mutational sequencing of HARs in an autism cohort revealed a variant in a HAR at the *CUX1* locus that was found to increase enhancer activity, leading to increased spine density with more stable synapses.^146^ This combination of comparative and human genetics is a powerful approach and provides compelling evidence that *CUX1* and its associated HAR may be important in the evolutionary differences in neuronal morphology.

To investigate mechanisms underlying differences in developmental tempo, recent studies have performed transplantations of human neurons and progenitors, revealing that the human-specific delay is maintained even when transplanted into the rodent cortex^147^ (Figure 3). Furthermore, comparisons of transplanted human and chimpanzee neurons revealed initially slower dendritic growth in human neurons, but a more complex final dendritic morphology.^148^ Additionally, electrophysiological recordings revealed slower acquisition of mature neuronal activity in human cells.

The genetic basis of this delay is still unclear, but recent comparative studies of human and rodent maturing neurons are beginning to highlight potential pathways. Two studies comparing human and mouse developmental clocks, one in the spinal cord and the other examining the segmentation clock, revealed a roughly 2.5-fold delay in human that was associated with slower protein turnover.^149,150^ This suggests that protein stability may be globally more delayed in human compared with mouse. This difference is intriguing, and while it is not enough to explain the overall difference between these species of more than 100-fold in brain developmental tempo and size, it may represent an important primate feature.

Recently, two sets of studies have compared maturing human and mouse neurons and revealed other possible global mechanisms involving metabolism and/or epigenetics. By comparing matched human and mouse developing neurons, Iwata et al. observed differences in mitochondrial morphology as neurons mature and a switch from glycolysis to oxidative phosphorylation, but in human this shift occurred more slowly.^151^ Human cells could be forced to undergo this metabolic switch earlier and thus this led to faster neuronal maturation, suggesting that metabolism can drive maturation speed. In another study, Ciceri et al. demonstrated an epigenetic barrier, involving the polycomb repressor complex, which prevents neuronal maturation and is gradually released during neuronal maturation.^152^ Premature release of this break, through the use of inhibitors of polycomb members, like EZH2, speeds up neuronal maturation, suggesting that this too can drive maturation speed. These studies again compared humans and mice, so it is still unknown whether such mechanisms could explain human-ape differences or rather represent primate-acquired features.

One possible evolutionary genetic mechanism for the delayed maturation is a set of duplication events in humans that led to the acquisition of three new versions of the *SRGAP2* gene, a regulator of neurite outgrowth and dendritic spine morphology.^153,154^ Two of these duplications, *SRGAP2B* and *SRGAP2C*, lead to functional proteins, in both cases a truncated version of the ancestral *SRGAP2*, and one of these (*SRGAP2B*) is located in the chromosome 1q21 region enriched in human duplicated genes.^155^ Overexpression of *SRGAP2C* in the mouse cortex inhibits *SRGAP2*, and inhibition in cultured mouse neurons or in the mouse cortex delays neuronal maturation and results in larger spines and increased spine density.^154^

### Neoteny and “bradychrony”

A common theme throughout developmental stages, from neuroepithelial transition to neuronal maturation, seems to be a human-specific delay in developmental tempo, often referred to as neoteny.^156^ Neoteny was originally coined in relation to the tadpole-like features of adult axolotl, thus referring to a retention of juvenile features^106^ (Figure 3). However, delayed development is not necessarily the same as retention of juvenile features. For example, sensory systems such as vision exhibit critical periods of experience-dependent plasticity that are prolonged in humans,^157^ but the olfactory system remains plastic throughout adulthood in mammals in general.^158^ The latter is an excellent example of neoteny, while the former is a delay. Neurons may take a long time to get there, but most do eventually reach a mature state. Because humans exhibit various other features reminiscent of a juvenile state (i.e., fine body hair), it is tempting to conflate the two, but in other primates where these juvenile characteristics are not retained, we still see a delayed neurodevelopmental tempo compared with rodents, suggesting that the two processes are not necessarily the same. The field is therefore in need of clarification. Neoteny is relevant for characteristics that remain immature, but for those traits where development completes but with a delayed tempo, “bradychrony” (meaning “slowed time”) may offer a more precise terminology. This distinction is important because it helps highlight the type of mechanism at play. For example, neoteny may not necessarily increase complexity, while the delay that comes with brady-chrony would enable a later acquisition of a more complex final product that nevertheless reaches a mature state (Figure 3).

## The Future of Human Brain Evolutionary Biology

### From correlation to causation

Although hints as to how human genetic changes may influence brain development and evolution are now coming to light, a complete mechanistic understanding is still lacking. For many observed traits, there is strong evidence that humans exhibit unique cellular and developmental traits, such as bradychrony and increased complexity. For some of these traits, there is also compelling genetic correlation with human-specific features, such as human-specific gene duplications and genetic variants in enhancer regions. In certain cases, there are even functional studies of the identified factors showing their roles in relevant cell types and stages. However, connecting the dots to a full understanding has not yet been done, mainly because the tools to do so are still so new.

To gain a complete mechanistic understanding, with the confidence to say that a particular genetic change is indeed responsible for a human-derived trait, it is necessary to not only perform functional studies of the encoded genes but also precise genetic manipulations that mimic as closely as possible the evolutionary genetic changes (Figure 4). For example, several of the genes unique to humans show correlated expression patterns during relevant stages when human-specific phenotypes arise and have been tested through overexpression studies in mice and even nonhuman primates. However, ectopic overexpression is not representative of the evolutionary change that has taken place in human evolution. Careful genetic manipulation of the endogenous locus in closely related species would enable examination of that particular genetic change in its *in situ* context, thus more accurately mimicking the actual acquired change. For example, studies with lentiviral overexpression of *ARHGAP11B* in the marmoset brain, and electroporation in human and chimpanzee organoids,^159^ provide strong evidence for a role in basal progenitor amplification. Such overexpression is informative but does not reflect the evolutionary change, as it involves introduction of an ectopic gene with multiple copies. Further studies in which the locus homologous to the human *ARHGAP11B* region is modified to carry the human gene would allow a clearer understanding of whether and how *ARHGAP11B* came to be an important novel factor in human brain evolution.

Other realms of human genetics, such as disease genetics^160^ and population genetics, can also provide important insight. Because of random genetic mutations that occur in a population, and the number of humans on earth, many of the regions of interest from an evolutionary perspective have had the chance for spontaneous mutations to arise within them. It is fairly safe to assume that a region that has been key for human brain evolution, if mutated, would cause a human disorder or, worse, lead to lethality. Thus, by examining those putative regions for mutations in human conditions, and/or examining whether healthy individuals exhibit mutations, one can further gain insight into whether and how a particular gene or enhancer may influence human brain development and evolution. This data, like perturbations in animal and *in vitro* models, provides compelling functional evidence for the importance of human-specific genetic changes.

The investigation of various HARs in an autism cohort^146^ provides strong indication that several of these regions do indeed play important roles in human brain development and function. Alternatively, investigation of genes or regions of interest in healthy human populations to test whether there is an absence of mutations can also strengthen the evidence for their role. For example, the Genome Aggregation Database (gnomAD) currently covers 76,215 genomes of healthy individuals from diverse ancestries, providing a quick and easy way to check whether a given gene is likely to be necessary for normal human development and physiology.^161^ A lack of individuals who are homozygous null for a particular gene is a strong indication that the gene is necessary. When it comes to recently acquired human-specific genes, there are 0 individuals in the database with homozygous null mutation in NOTCH2NLA, supporting its necessity. However, there are multiple homozygous null individuals for several of the other human-specific genes, including NOTCH2NLB, NOTCH2NLC, ARHGAP11B, SRGAP2B, and SRGAP2C. This certainly does not rule out their importance, as in many cases several duplicated versions exist, as in the case of NOTCH2NL and SRGAP2, suggesting redundancy. However, it is an important piece of evidence to consider and suggests that single-gene-knockout studies may not be all that informative in these cases.

### Questions that will be answered in the future

It is an exciting time in human evolutionary biology. The combination of cutting-edge methods promises to uncover truly mechanistic insight into how our brains have become so large and complex. It is only a matter of time before some of the biggest questions in human biology finally have answers. Here, I list a few predictions, which may or may not turn out to be true, but regardless represent areas of intense research that will no doubt yield exciting discoveries.

#### What enables the human brain to expand so much compared with the body?

The human brain is much larger than it should be compared with the body. Although much attention is focused on brain development and the neural progenitor behaviors that enable such a massive expansion, little-to-no attention has been paid to the rest of the body. Why is the human body not larger than a gorilla? Given our brain size, we should have a body size more than 3 times larger than a gorilla. Indeed, body growth in humans seems to be decreased compared with other mammals.^66^ An investigation of body growth could take advantage of *in vitro* models called gastruloids,^162,163^ which nicely model the posterior embryo and are composed of the various gastrulating germ layers of the body. Comparative studies of such models, in combination with studies of the brain, could answer this question.

#### How are brain and neural-crest evolution interconnected?

The brain and neural crest are inseparably linked. The two share a tissue of origin, the neuroepithelium, with their patterning set up even before the two separate, and they then continue to develop in close proximity and influence each other.^107^ Furthermore, the majority of craniofacial disorders also exhibit cognitive or neurodevelopmental defects.^164^ Finally, in parallel with evolutionary phenotypic changes in the brain, the *Homo sapiens* head and face has changed.^112^ This suggests that similar evolutionary mechanisms are at play in both. One possibility is the self-domestication hypothesis,^165^ which posits that, as humans, we have effectively domesticated ourselves by selecting for individuals with traits of domestication, such as decreased aggression and increased sociability. Wilkins et al.^166^ suggested such domestication is due to an evolutionary reduction in the neural crest, as exemplified by the changes in skull shape, tooth size, and a more flattened lower face and nose, as seen in domesticated animals. However, there are certain inconsistencies with this hypothesis,^167^ and while the neural crest likely does not explain all aspects of domestication, the fact that face shape in humans has changed along with changes in brain shape suggests the two may be linked. One possibility is that rather than the two changing in the same way (i.e., neural crest and brain reducing together), perhaps there is competition between the brain and neural crest. Given that the two originate from a common neuroepithelial origin, if less neuroepithelium delaminates to form neural crest, then more will be available to contribute to the brain. Thus, perhaps in humans, the balance has been shifted in favor of brain. Such a hypothesis would be consistent with the role of ZEB2 in delamination of neural crest cells and its delayed expression giving rise to a larger brain neuroepithelium in humans. However, much more research would be needed to test this hypothesis.

#### How is human bradychrony achieved to enable tissue expansion and increased cellular complexity?

The fact that developmental delay, or bradychrony, is a common feature of various stages of human neurodevelopment suggests that there may be a more global process at play. More specifically, rather than independently evolving a slower tempo for each developmental transition, a genetic change may have enabled bradychrony across transitions, from early neuroepithelial expansion to dendritic spine maturation and myelination. Recent studies comparing humans and mice are beginning to suggest that just such a global mechanism could be at play, pointing to protein turnover kinetics, metabolism, and epigenetics as potential players. Although these are exciting possibilities, it remains to be seen whether these processes explain the bradychrony that distinguishes humans from other apes and what the evolutionary genetic drivers of these processes may be. Future comparative ape studies and genetic investigations will no doubt answer this question.

#### What are the evolutionary genetic changes responsible for human-specific adaptations in the brain?

With the advent of CRISPR-Cas9 technology,^168^ and the development of elegant methods for genetic modification enabling even very large chromosomal substitutions,^169^ scientists are now in a position to be able to not only functionally perturb and test putative evolutionary genetic changes but also to even mimic those evolutionary changes in the endogenous genetic context. Future studies making use of scarless genome editing to introduce novel human genes and regulatory changes into nonhuman primate or ape cell lines will enable conclusions about causation to be drawn (Figure 4). These experiments are difficult, and will take time to be carried out properly, but with the availability of increased numbers of cell lines from various apes, moving beyond just humans and chimpanzees, such studies would provide the strongest evidence possible for which genetic changes are truly responsible for human-specific traits.

### Potential new directions

New omics technologies have opened up the ability to characterize and map the various cell types of the human brain and, importantly, to compare these with our closest living relatives. This area has changed our understanding of what defines a cell type and revealed a richer diversity of neuronal and glial types than was previously appreciated. Newer methods in spatial omics^170^ are already revealing important primate differences.^47,48^ Going forward, such comparisons within the primates and great apes, as well as new methods in single-cell epigenetics,^171^ will help uncover not only which cells are present but also where they are and how they got there. This is an exciting field, with ever more sensitive technologies on the horizon.

In the more distant future, it is likely that scientists will set their sights on broader evolutionary comparisons. Until recently, biology has necessarily had to focus on a limited number of model organisms. But, with the advent of induced pluripotent stem cells and next-generation sequencing, it is now becoming possible to investigate the genetics and biology of any mammal—and even, potentially, any vertebrate or invertebrate. Given the diversity of mammalian brain phenotypes and behaviors, it seems likely that *in vitro* models such as organoids will be applied to a greater diversity of animals. Already, rhino-induced pluripotent stem cells have been used to generate rhino brain organoids with promising structure and cell-type diversity.^172^ Such evolutionary comparisons could uncover new insights into the variety of ways the brain can expand, or evolutionary mechanisms for major differences in cellular diversity and cytoarchitecture, for example, the lack of granular layer IV in whales and dolphins.^173^ Examining the diversity of brain structures that nature has produced could reveal new ways of building neural circuitry.

Paleogenomics has now shed light on very recently acquired changes in the human genome. Although there are still a rather limited number of archaic hominin genomes available, comparison of modern human to Neanderthal or Denisovan genomes provides a window into our most recent genetic history.^174^ Unfortunately, it is impossible to know very much about phenotypic differences between the human and Neanderthal/Denisovan brain, thus it is difficult to go beyond speculation. However, cranial endocasts can provide some insight into overall brain shape (Figure 2). These have revealed that the Neanderthal brain was rather large and had a more oblong shape than a present-day human brain, with large occipital lobes and a sloping forehead.^61^ However, comparison with contemporaneous humans alive at the same time reveals little-to-no major differences in overall size or shape. In fact, the smaller size and more rounded shape of present-day human arose over the past 300,000 years of *Homo sapiens* evolution.^63^ Thus, such differences actually reflect an ancient-to-modern difference rather than a human-Neanderthal difference. It is also now believed that Neanderthals were advanced hominins capable of innovative tool use and artistic expression.^175^ Thus, while genetic differences between modern humans and Neanderthals are intriguing, studies of genetic changes within *Homo sapiens* over the past 300,000 years, and their potential effects on brain development, would be an exciting direction that has so far remained unexplored.

Although perhaps a long-shot, one exciting possible direction in the future will be the exploration of differences with our more distant hominin cousins and predecessors. Recently, a genome from a mammoth that was more than 1 million years old and preserved in the Siberian permafrost was sequenced,^176^ suggesting that it may even be possible to obtain genomic information from hominins older than ancient humans and Neanderthals. One species that we may be relatively more likely to have genetic access to is *Homo erectus. H. erectus* lived between about 2 million years ago and a little over 100,000 years ago.^177^ This puts its range within grasp of DNA sequencing. However, existing *H. erectus* remains are not preserved well enough to obtain intact genetic material because their range was primarily Africa and southern Asia. Nonetheless, there is some evidence that *H. erectus* may have ventured farther North because the Denisovan genome seems to contain traces of a more ancient hominin, potentially *H. erectus*.^178,179^ Because endocasts of *H. erectus* skulls reveal a much smaller brain size, a little over 2/3 the size of the *Homo sapiens* brain, genetic information would be highly informative in unraveling how human brain expansion came about.

Another area that will likely see further development is in modeling and applying functional neural circuits *in vitro* to the study of evolutionary differences. Studies to date have focused on cellular and developmental differences, but functional studies that move beyond simple electrophysiological metrics have not yet been done for evolutionary comparisons. Although multielectrode array recordings of *in vitro*-derived human and chimpanzee neurons revealed differences in neuronal firing rate,^148^ differences in network activity have not yet been described. Organoids have been shown to develop complex networks with long-range connections and hierarchical topology,^180^ and it would be exciting to examine whether differences in this topology are present between human and other ape organoids. For example, given the bradychronic nature of human neuronal maturation, one might expect an initially simpler network architecture in humans, but eventually a level of connectivity that surpasses the nonhuman ape to reach a more complex topology.

Finally, *in vitro* models open up a completely new way of doing evolutionary neuroscience that is not possible in living organisms: evolution in a dish. An exciting, albeit ambitious, possible future direction is engineering new phenotypes through directed evolution.^181^ For example, human neural stem cells could be subjected to mutagenesis and selected for increased proliferative capacity or their progeny selected for longer range connectivity. Such an approach could be applied as a simple screen but, likewise, directed evolution using successive rounds of mutagenesis could be applied by performing repeated reprogramming. This could be done in simple cultures but also even potentially more complex models like organoids. By taking evolution a step further, it may be possible to uncover new mechanisms and even engineer new cell types to accomplish tasks not yet possible in the human brain.

## Conclusions

The secrets of the human brain are still largely hidden, but we are now at a turning point in evolutionary neurobiology. The advent of a range of complementary, innovative new methods is opening doors that were previously closed to scientists. We can now not only observe and describe human-specific differences but also even perform genetic modifications to find out what evolutionary changes are really responsible for our large and complex brains. The challenge going forward will be prioritizing candidate genetic changes. There are many millions of human-acquired variants, the vast majority of which do nothing, and even most of those that do have a functional role have nothing to do with the brain. Narrowing in on those that are relevant to brain evolution is challenging, but elegant comparative omics and *in vitro* models will allow us to weed out those with no effect and to focus on those with stronger evidence for a functional role. The next 50 years will be exciting, and although the door to understanding the secrets of human brain evolution is only slightly ajar, it is about to be swung wide open.
